# Mutations in Diphosphoinositol-Pentakisphosphate Kinase PPIP5K2 are associated with hearing loss in human and mouse

**DOI:** 10.1371/journal.pgen.1007297

**Published:** 2018-03-28

**Authors:** Rizwan Yousaf, Chunfang Gu, Zubair M. Ahmed, Shaheen N. Khan, Thomas B. Friedman, Sheikh Riazuddin, Stephen B. Shears, Saima Riazuddin

**Affiliations:** 1 Laboratory of Molecular Genetics, Department of Otorhinolaryngology-Head & Neck Surgery, School of Medicine University of Maryland, Baltimore, MD, United States of America; 2 Signal Transduction Laboratory, National Institute of Environmental Health Sciences, National Institutes of Health, Research Triangle Park, NC, United States of America; 3 Laboratory of Neurogenetics and Regenerative Medicine, Department of Otorhinolaryngology-Head & Neck Surgery, School of Medicine University of Maryland, Baltimore, MD, United States of America; 4 National Center for Excellence in Molecular Biology, University of the Punjab, Lahore, Pakistan; 5 Section on Human Genetics, Laboratory of Molecular Genetics, National Institute on Deafness and Other Communication Disorders, National Institutes of Health, Bethesda, MD, United States of America; 6 Shaheed Zulfiqar Ali Bhutto Medical University, Pakistan Institute of Medical Sciences, Islamabad, Pakistan; Tel Aviv University, ISRAEL

## Abstract

Autosomal recessive nonsyndromic hearing loss is a genetically heterogeneous disorder. Here, we report a severe-to-profound sensorineural hearing loss locus, *DFNB100* on chromosome 5q13.2-q23.2. Exome enrichment followed by massive parallel sequencing revealed a c.2510G>A transition variant in *PPIP5K2* that segregated with DFNB100-associated hearing loss in two large apparently unrelated Pakistani families. PPIP5Ks enzymes interconvert 5-IP7 and IP8, two key members of the inositol pyrophosphate (PP-IP) cell-signaling family. Their actions at the interface of cell signaling and bioenergetic homeostasis can impact many biological processes. The c.2510G>A transition variant is predicted to substitute a highly invariant arginine residue with histidine (p.Arg837His) in the phosphatase domain of PPIP5K2. Biochemical studies revealed that the p.Arg837His variant reduces the phosphatase activity of PPIP5K2 and elevates its kinase activity. We found that in mouse inner ear, PPIP5K2 is expressed in the cochlear and vestibular sensory hair cells, supporting cells and spiral ganglion neurons. Mice homozygous for a targeted deletion of the *Ppip5k2* phosphatase domain exhibit degeneration of cochlear outer hair cells and elevated hearing thresholds. Our demonstration that PPIP5K2 has a role in hearing in humans indicates that PP-IP signaling is important to hair cell maintenance and function within inner ear.

## Author summary

Exome sequencing coupled with homozygosity mapping was used to identify a missense variant [c.2510G>A; p.(Arg837His)] in PPIP5K2 at the *DFNB100* locus that is associated with nonsyndromic, prelingual sensorineural deafness in two large consanguineous Pakistani families. PPIP5Ks are pivotal enzymes for regulating inositol pyrophosphate (PP-IP) turnover. Biochemical analyses revealed that, compared to wild type human PPIP5K2, the PPIP5K2^R837H^ variant exhibited lower phosphatase activity and higher kinase activity, indicating that it promotes increased metabolic flux from 5-IP7 to IP8 *in vivo*. In rodent inner ears, PPIP5K2 immunoreactivity was observed in the cochlear and vestibular hair cells, supporting cells, and spiral ganglion neurons. Mouse mutants homozygous for the targeted deletion of *Ppip5k2* phosphatase domain exhibit degeneration of cochlear outer hair cells and progressive hearing loss. Our work provides the first description of any amino acid variant of PPIP5K2 that is both functionally-significant and associates with a human disorder. The ‘futile cycling’ of the kinase/phosphatase activity of PPIP5K2 makes inner ear function particularly susceptible to even minor changes in the phosphatase activity of PPIP5K2. We have shown that a pathogenic variant in *PPIP5K2* is associated with hearing loss in humans. Thus, PPIP5K2 is given new clinical significance by our observations.

## Introduction

Hearing loss (HL) is a heterogeneous neurosensory deficiency that occurs at all ages with varying severities, affecting 1 in 500 newborns and >360 million people worldwide [1]. Many forms of HL are inherited, and ~400 syndromic forms occur with linked medical comorbidities. Genetically complex non-syndromic recessively inherited HL (NSRHL) comprises ~75% of hereditary deafness [2]. RNA profiling studies revealed expression of 18,133 genes in the organ of Corti (OC) hair and supporting cells [3], underscoring the complexity of inner ear development and function. Elucidating the roles of each of these genes by conventional methods would require generating mouse or other animal models for each. By contrast, genetic studies of human families segregating HL has significantly helped in overcoming the barriers of sheer scale, functional redundancy, and non-essential roles of some genes. Discovering a variant associated with human deafness rules out functional redundancy at least in the auditory system. Genetic and functional studies of the deafness associated proteins have been pivotal in elucidating various molecular networks essential for hearing [4–6].

Here, we describe two large consanguineous Pakistani families segregating NSRHL that we associate with a missense variant [p.(Arg837His)] in the PPIP5K2 (E.C. 2.7.1.155). PPIP5Ks are enzymes that interconvert 5-IP7 and IP8, two key members of the inositol pyrophosphate (PP-IP) cell-signaling family [7]. Humans express two PPIP5K enzymes, PPIP5K1 (160 kDa) and PPIP5K2 (138 kDa) [8]. These are large enzymes that contain a kinase domain that phosphorylates 5-IP7 to IP8, and a separate phosphatase domain that dephosphorylates IP8 back to IP7. Little is known concerning how these competing reactions are coordinated. Nevertheless, PPIP5Ks regulate the levels of PP-IPs, which impact endocytosis, vesicle trafficking, apoptosis, spermatogenesis, secretion of insulin from pancreatic β cells, and DNA repair [9–11]. IP8 is also a sensor of extracellular inorganic phosphate [8] and regulates bioenergetic homeostasis [12].

Our *in vitro* biochemical studies indicate that the p.Arg837His deafness-associated variant of PPIP5K2 has reduced phosphatase activity and increased kinase activity. As a consequence, the variant has the capacity to synthesize more of the IP8 signal *in vivo*. An elevated cellular production of IP8 can be recapitulated in mice harboring a homozygous deletion of the phosphatase domain of PPIP5K2, thereby unmasking higher kinase activity. These animals exhibited degeneration of cochlear outer hair cells and progressive hearing loss. To the best of our knowledge, this is the first study demonstrating the necessity of PP-IP metabolism for inner ear development and hearing function.

## Results

### Families PKDF041 and PKDF751 have prelingual profound HL

**Fig 1:**
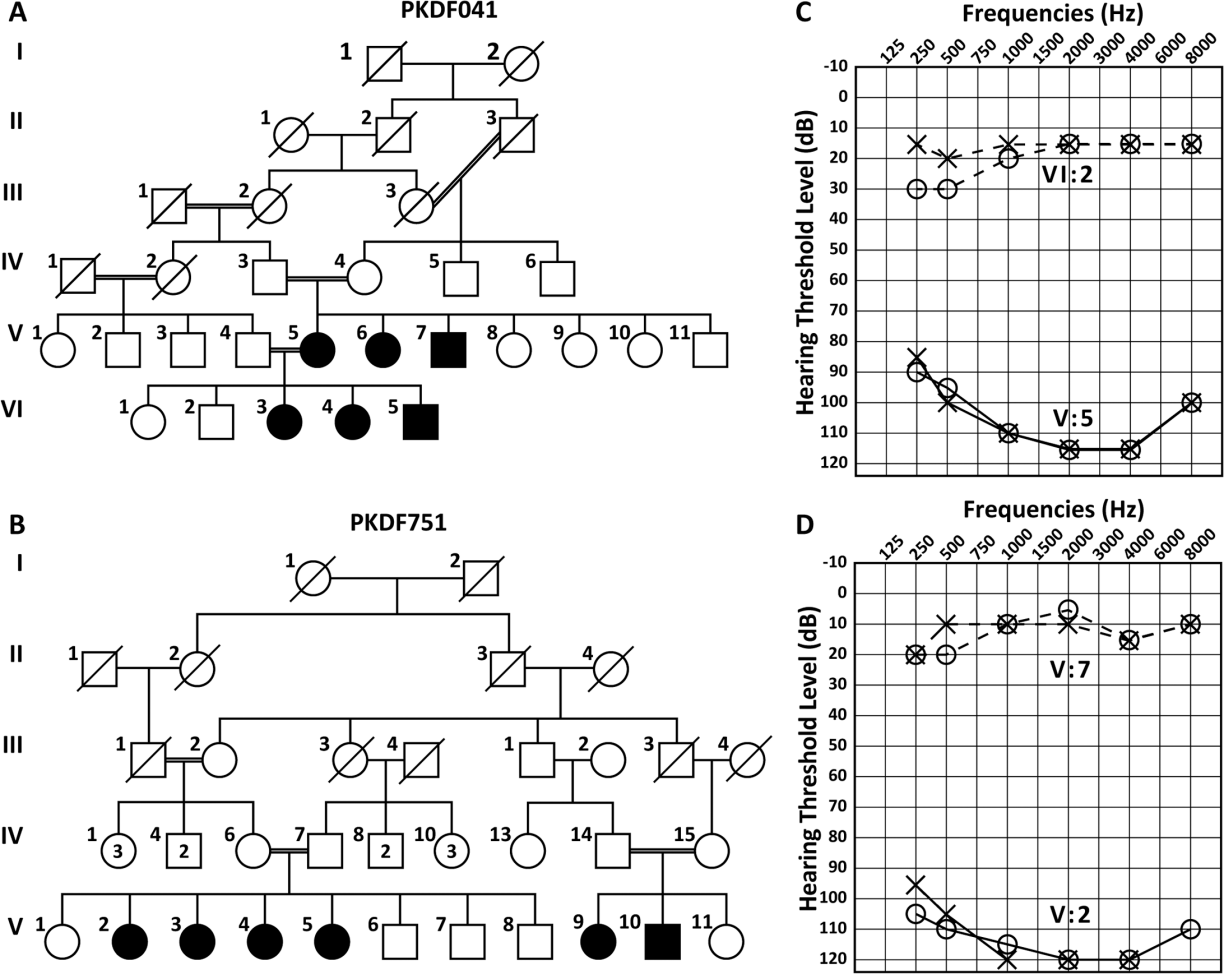
Pedigrees of families segregating DFNB100 deafness and representative pure-tone audiograms. (A, B) Squares and circles denote male and female family members, respectively. Filled symbols represent affected individuals. (C) Pure tone air conduction thresholds for family PKDF041 individuals VI:2 (9 yo male), and V:5 (42 yo male). Individual VI:2 hearing thresholds were within normal range, while individual V:5 had profound HL in both ears. Right ear air conduction: O; Left ear air conduction: X. (D) Pure tone air conduction thresholds for family PKDF751 individuals V:2 (23 yo female) revealed profound HL in both ears. In contrast, individual V:7 (20 yo male) had hearing threshold within normal range.

Two large apparently unrelated families PKDF041 and PKDF751 segregating NSRHL were enrolled from Punjab province of Pakistan (Fig 1A and 1B). Affected individuals of both families have prelingual bilateral severe to profound sensorineural hearing loss (Fig 1C and 1D). According to family history no hearing was noted since birth in all of the affected individuals. Tympanometry of three of the affected [V:2 (19 yrs), V:3 (23 yrs), V:5 (26 yrs)], and one normal hearing individual (V:7; 22 yrs) of family PKDF751 revealed no abnormalities of the tympanic membrane or middle ear. However, these affected individuals (V:2, V:3, V:5) failed a transient evoked otoacoustic emission test, which is indicative of defective outer hair cell function. Romberg and tandem gait tests did not reveal any overt vestibular dysfunction in affected individuals of both families. Funduscopy examinations of affected individuals of PKDF041, V:5 (42 years), VI:3 (16 years), VI:4 (10 years) and VI:5 (20 years) revealed no evidence of retinitis pigmentosa. In addition, ERG a- and b-waves amplitudes for the affected individuals [V:5 (42 yrs), V:7 (30 yrs)] of PKDF041, were normal [13], further excluding a retinal degeneration phenotype.

### Linkage analysis mapped a new deafness locus, *DFNB100* on chromosome 5q

Linkage analysis of NSRHL segregating in family PKDF041 with STR markers across the genome, previously revealed linkage to *DFNB49* locus on chromosome 5, with a maximum two-point lod score (Zmax) of 4.44 (θ = 0) for marker *D5S2055* [13]. Subsequently, mutations in *TRIC* were identified as the cause of *DFNB49*-linked HL [14, 15]. Full sequencing of *TRIC* in the genomic DNA from two affected individuals from the PKDF041 family along with a normal hearing sibling did not reveal any pathogenic variants, suggesting there is an additional gene in the PKDF041 linkage interval in which a pathogenic variant is associated with deafness.

Subsequently, genome wide linkage analysis in DNA samples from a second family, PKDF751, revealed a large region of homozygosity shared among the affected individuals on human chromosome 5q12.2-q23.3, which completely overlaps with the linkage interval defined by family PKDF041 (S1 Fig). Similar to family PKDF041, mutation in the protein coding exons, non-coding exons or in the splice junctions of *TRIC* were not detected in the affected individuals of family PKDF751. Therefore, the HUGO nomenclature committee assigned the *DFNB100* designation for the locus defined by families PKDF041 and PKDF751 (S1 Fig). Besides *TRIC*, other candidate genes include *OCLN*, *SHROOM1*, *GPR98*, *KCNN2* and *SLC12A2* (S1 Fig) [16–21]. However, sequencing of all these genes did not reveal a potentially pathogenic variant among the affected individuals of families PKDF041 and PKDF751. Rather than continue hierarchical sequencing of candidate genes based on function or expression in the inner ear, we employed an exome sequencing approach to identify a mutant variant responsible for deafness at the *DFNB100* locus.

### A missense variant in *PPIP5K2* is associated with DFNB100-deafness

**Fig 2:**
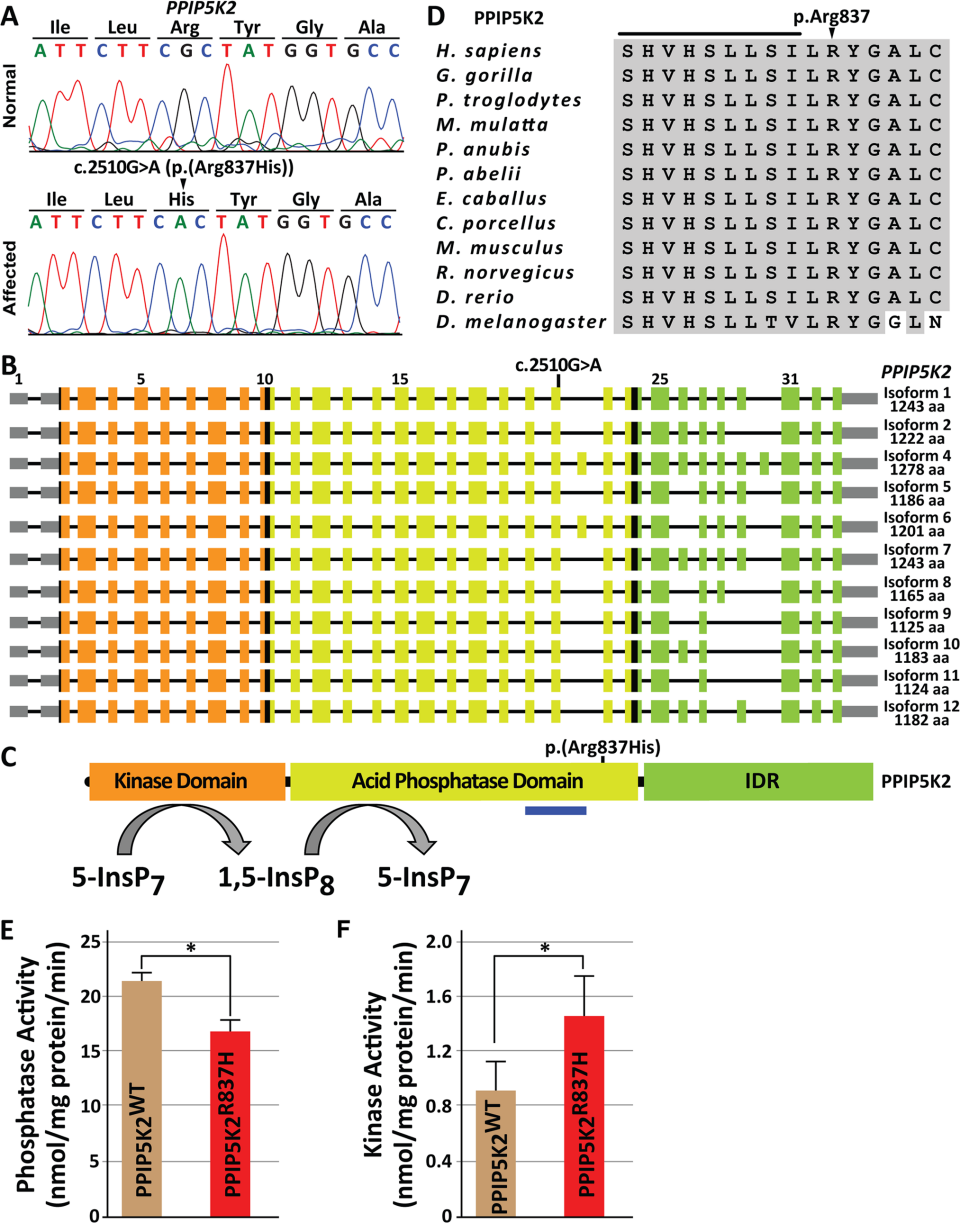
Point mutation in *PPIP5K2* is associated with DFNB100 hearing loss (HL). (A) Nucleotide sequence chromatograms of selected region of *PPIP5K2* exon 21 from homozygous WT (normal) and affected individuals harboring c.2510G>A [p.(Arg837His)] mutation segregating in DFNB100 families. (B) The human *PPIP5K2* gene has 33 exons. Alternate splicing of *PPIP5K2* gives rise to many different transcripts that are predicted to affect the composition of the non-catalytic intrinsically disordered region (IDR). The non-coding 5’ and 3’ UTRs are gray rectangles while sequences encoding the kinase, phosphatase (pase) and IDR domains, and other coding regions of exons are denoted by orange, light green, dark green, and black boxes, respectively. The nucleotide and amino acid variants of *PPIP5K2* are also shown. The blue bar under the phosphatase domain indicates the antigenic region of the commercially available polyclonal antibodies. (C) Schematic representation of PPIP5Ks biochemical function. Arrows indicate direction of the reaction. (D) PPIP5K2 amino acid conservation from eleven vertebrates and one non-vertebrate (Drosophila). Conserved amino acids are shaded in gray, while the p.Arg837 amino acid affected due to c.2510G>A variant, identified in our DFNB100 families, is noted with an arrow head. (E) The phosphatase activities of FLAG-tagged WT PPIP5K2 (21.5 ± 1.5 nmol/mg protein/min) and PPIP5K2^R837H^ (16.9 ± 2.1 nmol/mg protein/min) were determined as described in the Methods section (*p<0.05, n = 5; mean ± SE). (F) The kinase activities of FLAG-tagged WT PPIP5K2 (0.9 ± 0.21 nmol/mg protein/min) and PPIP5K2^R837H^ (1.44 ± 0.29 nmol/mg protein/min) were determined as described in the Methods Section (*p<0.03, n = 5; mean ± SE).

To enrich and capture coding regions, we used genomic DNA samples from one affected individual and one unaffected control from family PKDF751, and performed massively parallel sequencing of the exome. Copy number variants (CNVs) analysis did not reveal any indels (≥50bp) associated with deafness within *DFNB100* linkage interval. Our filtering of the exome sequencing data revealed three genes with predicted pathogenic changes within DFNB100 linkage interval (S1 Table). Sanger sequencing revealed a single point mutation (c.2510G>A) in the *PPIP5K2* gene (NCBI RefSeq NM_001276277) segregating with HL in family PKDF751 (Fig 2A), which is predicted to replace an evolutionarily-conserved arginine with histidine at residue 837 (NP_001263206; Fig 2B, S2 Table). Sanger sequencing of all coding and non-coding exons of *PPIP5K2* in the DNA samples from family PKDF041 confirmed association of the same c.2510G>A variant with deafness. SNPs linked to *PPIP5K2* were genotyped in affected individuals of the PKDF041 and PKDF751 families, and the flanking haplotype was consistent with a founder effect for c.2510G>A variant (S3 Table). We did not detect this variant in 180 ethnically-matched Pakistani control samples, nor in small publicly-available databases (1000 Genome [22], or NHLBI_EP [23]). At present, the c.2510G>A variant of *PPIP5K2* is at a low frequency (0.000146) in the ExAC database [24], and thus c.2510G>A is not a common polymorphism.

### The PPIP5K2^R837H^ variant modifies the catalytic activities of the enzyme

Human *PPIP5K2* encodes at least eleven alternatively spliced isoforms, all of which are affected by the p.(Arg837His) substitution identified in the two DFNB100 HL families (Fig 2B). PPIP5K2 has three distinct functional regions: a kinase domain, a phosphatase domain and an intrinsically disordered region that may mediate protein-protein interactions (Fig 2B). The Arg837His substitution is located in the phosphatase domain (Fig 2C). Human PPIP5Ks is a mutually competitive kinase and phosphatase that interconverts PP-IPs (Fig 2C). *In silico* analyses predict that the p.Arg837His residue is likely of structural and/or functional importance (S2 Table). Indeed, Arg837 is conserved throughout the animal kingdom (Fig 2D), and lies nine residues C-terminal to a conserved histidine residue that is catalytically-essential for IP8 phosphatase activity in a yeast orthologue of PPIP5K2 [25]. Thus, p.Arg837 is a candidate for binding the negatively charged PP-IP substrate. The substitution of histidine would be less effective in this role as its side chain is less polar at physiological pH [26]. Another possibility is that p.Arg837 acts in a structurally-stabilizing salt bridge, which could be disrupted by substitution with His [27].

To pursue these ideas, we first assayed directly the impact of the p.Arg837His variant upon the phosphatase activity of PPIP5K2 *in vitro*, using recombinant, FLAG-tagged WT and PPIP5K2^R837H^ which were expressed in HEK293 cells and then immuno-affinity purified (Fig 2E). The IP8 phosphatase activity of the PPIP5K2^R837H^ protein was reduced by 21% (*p< 0.05) as compared to the WT protein (Fig 2E). PPIP5K2 interconverts 5-IP7 to IP8 through the actions of mutually competing phosphatase and kinase domains. Thus, we hypothesized based upon the *in vitro* kinase assays of 5-IP7 phosphorylation that PPIP5K2^R837H^ protein would accumulate more IP8 than WT PPIP5K2. That proposal presumes that a potentially higher level of accumulation of IP8 by the PPIP5K2^R837H^ protein is not negated by an increased rate of IP8 dephosphorylation. However, that self-correction did not occur. Assays that used PPIP5K2^R837H^ accumulated 60% higher levels of IP8 compared to assays that contained WT protein (Fig 2F). The proportionately higher effect of the p.Arg837His variant upon the kinase activity may reflect an impact upon conformational coupling between the two domains [8], in addition to the reduction in phosphatase activity. Note that, *in vitro*, the phosphatase and kinase domains of PPIP5K2 can also be shown to interconvert IP6 and 1-IP7 [12]. However, these particular reactions proceed relatively slowly [12]; their general biological significance is in doubt, especially as levels of 1-IP7 in mammalian cells are virtually undetectable [11].

### *Ppip5k2* is ubiquitously expressed in mice

**Fig 3:**
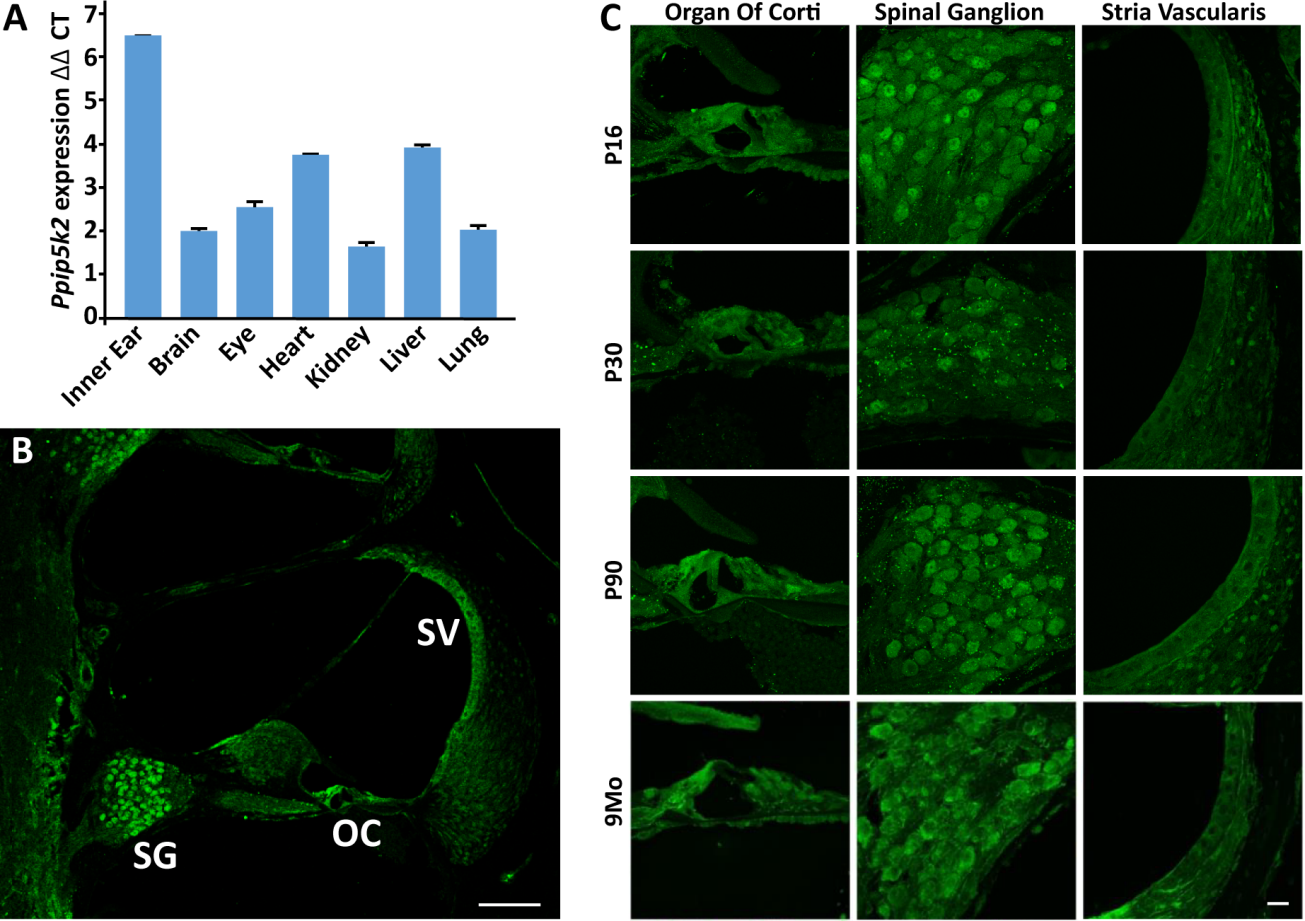
PPIP5K2 localized in sensory and non-sensory cells of mouse inner ear. (A) Expression of *Ppip5k2* in adult (P150) mice, normalized against *Gapdh* (ΔCT) and *Ppip5k1* expression (ΔΔCT). (B) Cross-section through one of the coils of inner ear showing diffuse cytoplasmic immunolabeling of PPIP5K2 throughout the cochlear duct, including spiral ganglion neurons (SG), organ of Corti (OC), and stria vascularis (SV). (C) Expression of PPIP5K2 persists at the ages tested in WT mice, from early postnatal day P16, up to 9 months of age. Scale bars: 100μm (panel A), and 20μm (panel C).

Our analysis by real-time PCR showed *Ppip5k2* and *Ppip5k1* to be widely expressed in mouse tissues, including inner ear (Fig 3A, S3A Fig). Transcriptome analysis indicates that *Ppip5k2* is the major *Ppip5k* gene to be expressed in the mouse cochlear and vestibular sensory hair cells, supporting cells as well as in ganglion neurons, while levels of *Ppip5k1* expression in these cells are up to 100-fold lower (gEAR, SHIELD) [28]. Similarly, PPIP5K2 immunoreactivity, assessed using an antibody directed against a region within the phosphatase domain (Fig 2B), was observed in all these cell types at various developmental stages (Fig 3). Moreover, all three layers of the stria vascularis (marginal, intermediate and basal cells) exhibited PPIP5K2 immunoreactivity (Fig 3B and 3C). The distribution of PPIP5K1 immunoreactivity within the inner ear, assessed using isoform-specific antibodies (S2 Fig), exhibited an almost identical expression profile to that of PPIP5K2 (S3B–S3E Fig).

### Disruption of murine *Ppip5k2* promotes auditory deficit and hair cell degeneration

**Fig 4:**
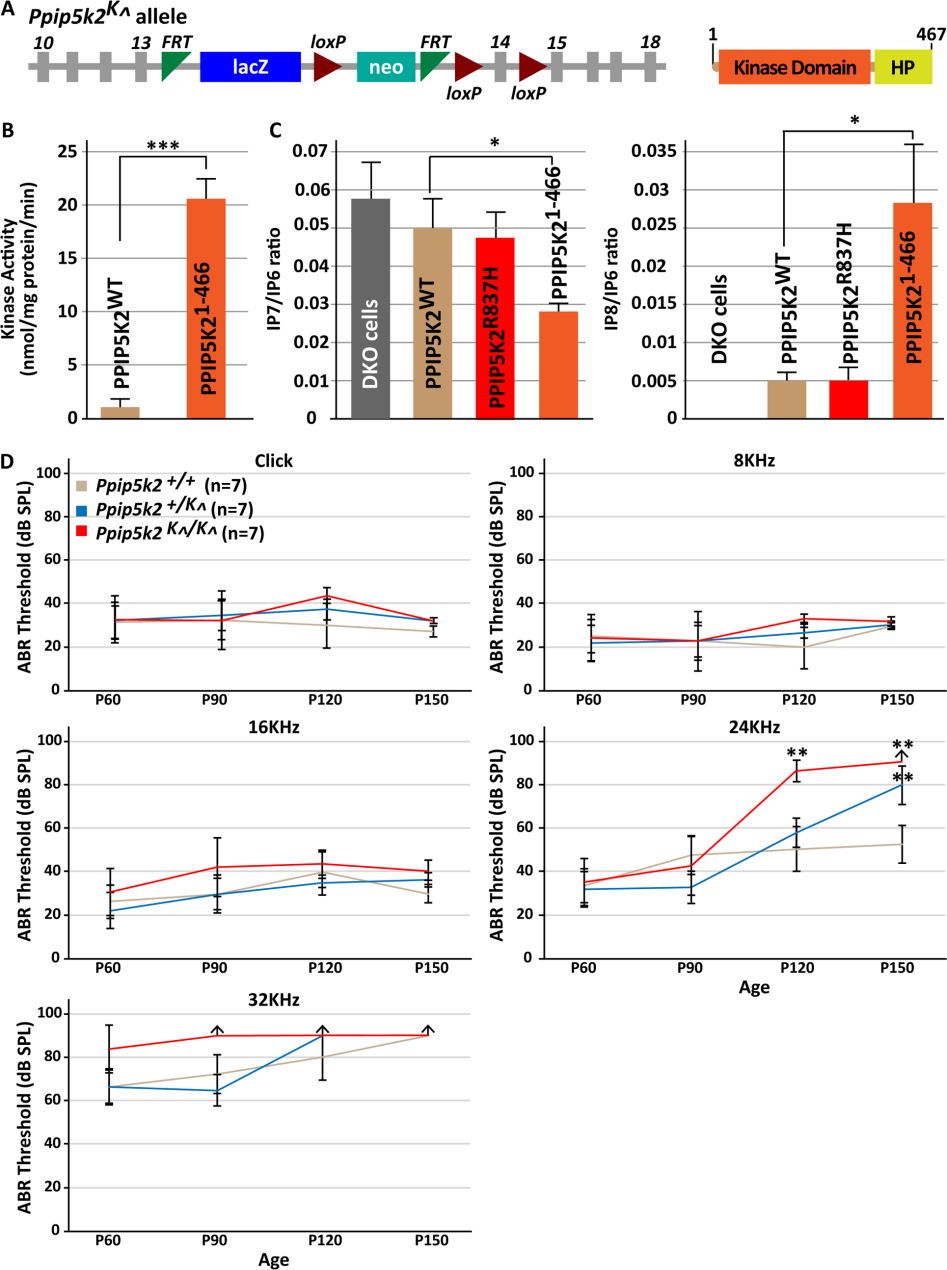
*Ppip5k2*^*K*^*^*^*/K*^*^* mice exhibit late onset high-frequency progressive HL. (A) Schematic of the *Ppip5k2*^*K*^*^* allele (left), and resulting truncated PPIP5K2 protein (right) with a truncated (only the first 82 residues) of the histidine acid phosphatase motif (HP). (B) The kinase activities of FLAG-tagged WT (0.9 ± 0.21) and PPIP5K2^1-466^ (20.3 ± 1.0), which has kinase and HP motif but no phosphatase activity, and corresponds to the protein expressed by the mouse *Ppip5k2*^*K*^*^* allele (*p<0.001, n = 5; mean ± SE). (C) Levels of IP6, IP7 and IP8 were determined by HPLC in *PPIP5K1/2* double knockout (DKO) HEK293 cells transfected with various *PPIP5K2* constructs. Bar graphs display mean ± SE (n = 6, except PPIP5K2^1-466^ n = 5). IP7/IP6 ratio in DKO cells (0.057 ± 0.009, n = 6), DKO cells transfected with FLAG-tagged WT (0.05 ± 0.006), PPIP5K2^R837H^ (0.048 ± 0.006) and PPIP5K2^1-466^ (0.028 ± 0.0029; *p<0.011). Shown also IP8/IP6 ratio (*p<0.023) in DKO cells (0 ± 0), cells transfected with FLAG-tagged WT (0.0049 ± 0.0012), PPIP5K2^H837R^ (0.0051 ± 0.0014) and PPIP5K2^1-466^ (0.028 ± 0.008) constructs. (D) Average click and pure tone-evoked ABR thresholds (dB SPL) in *Ppip5k2*^*+/+*^, *Ppip5k2*^*+/K*^*^*, and *Ppip5k2*^*K*^*^*^*/K*^*^* at different time points (P60, P90, P120, P150) were assessed (n = 7; mean ± SEM). Raised mean ABR thresholds at 32 kHz were detected as early as P60 in *Ppip5k2*^*K*^*^*^*/K*^*^* mice and continued to increase with age. At P120 and P150, the *Ppip5k2*^*K*^*^*^*/K*^*^* mice had profound HL at higher frequencies [24 kHz (**p<0.001) and 32 kHz]. Also, *Ppip5k2*^*+/K*^*^* mice exhibited elevated hearing thresholds loss at higher frequencies [24 kHz (*p<0.05) and 32 kHz]. These results suggest impaired PPIP5K2 function in mouse is associated with late onset high-frequency progressive HL. However, as anticipated, due to age-related hearing loss in mice on a C57BL/6 genetic background, the ABR thresholds at 32 kHz were also elevated in WT mice at P120 and P150.

To determine the role of PPIP5K2 in the inner ear, we obtained a mouse from the Knockout Mouse Phenotyping Program (KOMP) that has a gene trap cassette with a LacZ reporter in the intron between exons 13 and 14 of *Ppip5k2* (Fig 4A). The gene trap leads to the translation of a protein in which the N-terminal kinase domain is intact, but the adjoining phosphatase domain is truncated to just 84 residues (Fig 4A), which is insufficient to encode phosphatase activity [29, 30]. To study the impact upon the enzymatic properties of the protein product of the targeted allele, we prepared a recombinant, C-terminally truncated human PPIP5K2 (PPIP5K2^1-466^). This is equivalent to the murine protein that is expected to be expressed by the *Ppip5k2*^*K*^*^* allele. The shorter human protein converted 5-IP7 to IP8 at a 23-fold higher rate than that catalyzed by the kinase domain of the full-length human PPIP5K2 (Fig 4B). We therefore named the murine allele as *Ppip5k2*^*K*^*^*.

It is impractical to assay the impact of the *Ppip5k2*^*K*^*^* allele upon PP-IPs *in vivo*, due to PP-IPs sub- to low-micromolar levels, and the relative insensitivity of mass assays for these molecules. Instead, we used [^3^H]-inositol radiolabeled *PPIP5K*^-/-^ HEK293 cells [12], as a host for over-expression of either WT human PPIP5K2 or the truncated PPIP5K2^1-466^ mutant. The latter supported the synthesis of 4-fold (n = 3) higher cellular IP8 levels than those sustained by the WT protein (Fig 4C), despite the expression level of truncated PPIP5K2^1-466^ being much lower than that of WT PPIP5K2 (S4 Fig). Based on these findings, we conclude that the *Ppip5k2* mutant mice likely expresses a “hyper kinase” activity. Note that the changes in cellular levels of IP7 and IP8 resulting from over-expression of PPIP5K2^R837H^ in *PPIP5K*^-/-^ HEK293 cells [12] were similar to those observed after over-expression of WT PPIP5K2 (Fig 4C). The catalytic differences between these particular enzymes are less prominent (Fig 2E and 2F), making it harder to detect their differential impact upon PP-IP turnover in intact cells. PP-IP levels will also be influenced by cell-type dependent differences in expression of other PP-IP kinases and phosphatases.

In *Ppip5k2*^*K*^*^*^*/K*^*^* mice, we observed a significant upregulation of *Ppip5k1* message in several tissues at P150, including a nearly 3-fold elevation in the inner ear (S3F Fig). Any increased expression of *Ppip5k1* can be predicted to further enhance the synthesis of IP8 [12]. However, the metabolic consequences of this effect are likely minor in the inner ear, where relative levels of *Ppip5k1* are 100-fold less than those of *Ppip5k2* (see above).

**Fig 5:**
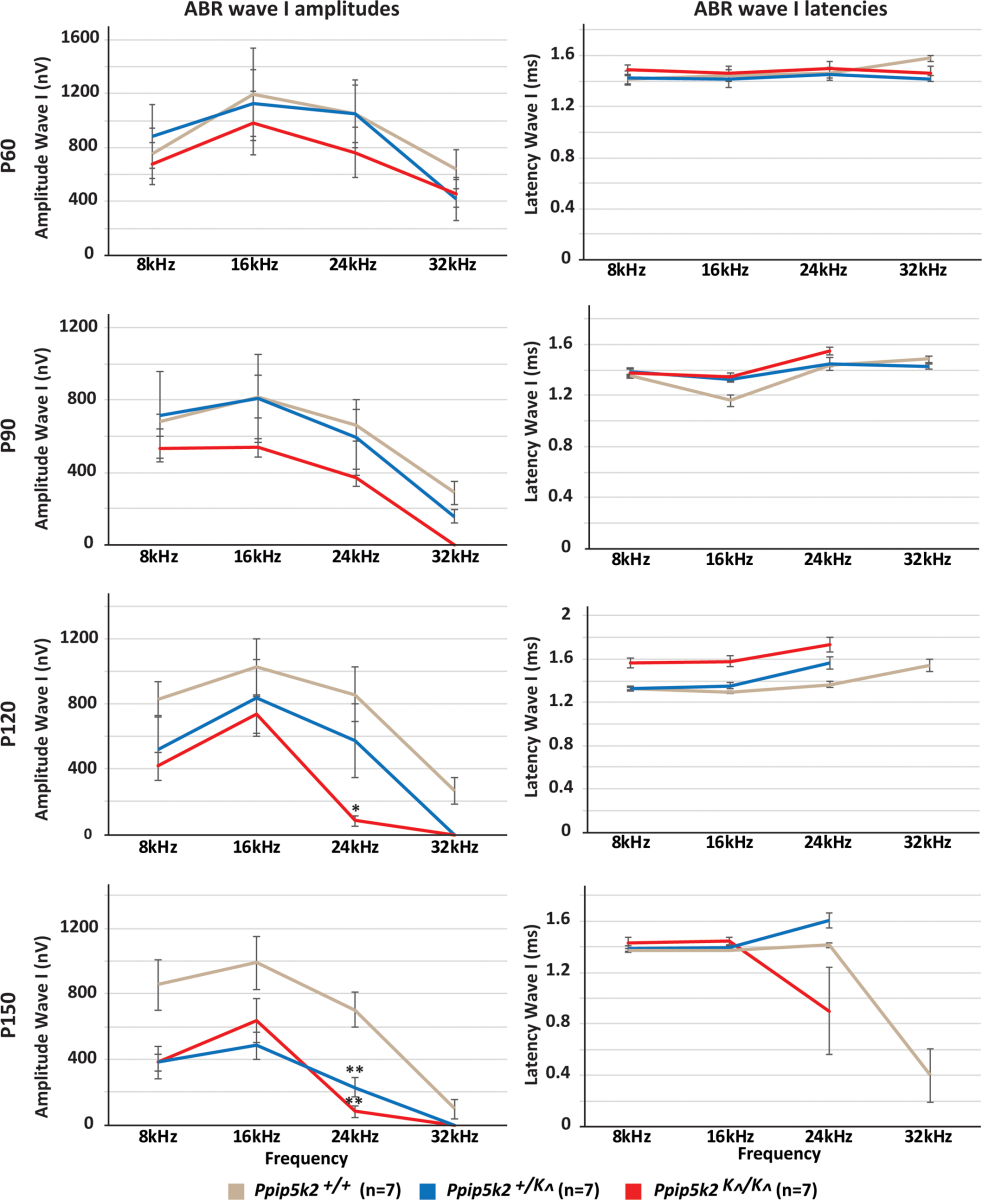
*Ppip5k2*^*K*^*^*^*/K*^*^* mice exhibit late onset high-frequency decline in ABR wave I amplitudes. Average pure tone-evoked ABR wave I amplitudes and latencies in *Ppip5k2*^*+/+*^, *Ppip5k2*^*+/K*^*^*, and *Ppip5k2*^*K*^*^*^*/K*^*^* at different time points (P60, P90, P120, P150) were assessed (n = 7; mean ± SEM). Significantly reduced wave I amplitudes at 24KHz were detected at P120 in *Ppip5k2*^*K*^*^*^*/K*^*^* mice (*p < 0.01). At P150 both the homozygous and heterozygous *Ppip5k2*^*K*^*^* mice had significantly reduced wave I amplitudes at 24KHz mice (**p < 0.05). These results suggest impaired late onset auditory neuronal function in PPIP5K2 mutant mice.

To characterize inner ear function, we measured auditory-evoked brainstem responses (ABR) in *Ppip5k2*^*K*^*^*^*/ K*^*^* and WT mice at various developmental stages (S5 Fig). No statistically significant difference in hearing thresholds was observed in *Ppip5k2*^*K*^*^*^*/ K*^*^* as compare to WT at postnatal day 60 (P60), when tested for broad band clicks as well as pure tone burst of frequencies 8, 16, and 24 kHz (Fig 4D). However, *Ppip5k2*^*K*^*^*^*/K*^*^* mice had slightly elevated, but not statistically significant, thresholds at 32 kHz compared to WT mice (Fig 4D, top left). The difference progressively increased with age. At P90, the threshold at 32 kHz of *Ppip5k2*^*K*^*^*^*/K*^*^* mice were statistically significantly (p<0.05) elevated as compare to WT (Fig 4D, top right). At P120 and P150, significantly (p<0.001) higher threshold was observed at 24 kHz as well in *Ppip5k2*^*K*^*^*^*/K*^*^* (Fig 4D, bottom), which suggest progression of hearing loss from base towards middle cochlear turn. The *Ppip5k2*^*+/K*^*^* mice at P150 also exhibited significantly higher threshold (p < 0.05) at 24 kHz, which suggest that reduced amount of WT protein is also not sufficient to maintain hearing function in older mice. As anticipated, considering the C57BL/6 genetic background, which is susceptible to age-related HL [31, 32], at P120 and P150, the thresholds for 32 kHz were observed to be elevated in both genotypes. We also compared the ABR wave I amplitudes and latencies in control and *Ppip5k2* ^*K*^*^* mutant mice (Fig 5). Significant reduction in wave I amplitudes at higher frequency (24kHz) was observed in older homozygous mutant mice. Overall, ABR auditory data from mice are consistent with the conclusion that elevated PPIP5K2 kinase activity is a causal factor for inherited auditory defects, although the deficit in the *Ppip5k2*^*K*^*^*^*/K*^*^* mice is less severe than that observed in humans homozygous for the p.(Arg837His) substitution.

**Fig 6:**
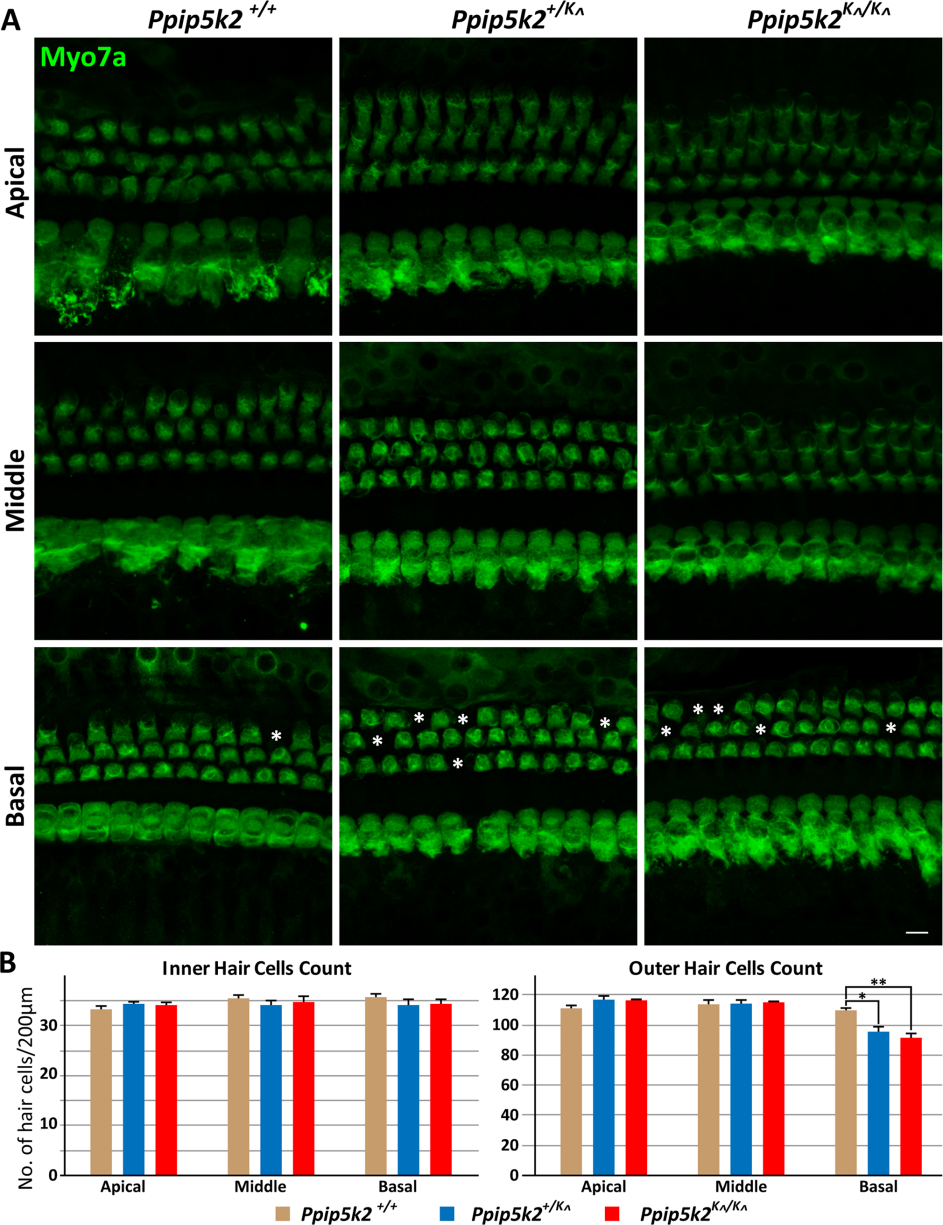
*Ppip5k2*^*K*^*^* mice exhibit late onset degeneration of OHCs in the basal cochlear turn, which detects high frequency sound. (A) Confocal images of the organ of Corti, labeled with antisera against MYO7A, a hair cell marker, at the apical, middle and basal turns of the cochlea in *Ppip5k2*^*+/+*^, *Ppip5k2*^*+/K*^*^*, and *Ppip5k2*^*K*^*^*^*/K*^*^* mice at P150. In WT mice, the position of dead OHCs are marked with asterisks (*), observed only in the basal turn. In contrast, significantly higher number of OHCs were missing in *Ppip5k2*^*+/K*^*^*, and *Ppip5k2*^*K*^*^*^*/K*^*^* mice, which correlates with the high-frequency HL observed in these mice. Scale bar: 20μm. (B) Inner hair cells and outer hair cells were quantified in *Ppip5k2*^*+/+*^, *Ppip5k2*^*+/K*^*^*, and *Ppip5k2*^*K*^*^*^*/K*^*^* mice at P150. For quantification purposes, organ of Corti were isolated from three different mice of each genotype, and the hair cells were counted in 200μm stretches of apical, middle and basal coil regions. No significant difference was observed for the number of IHCs. Whereas in the case of OHC number (mean ± SEM), a statistically significant (*p<0.05, **p<0.01) decrease in OHCs were observed in the basal coil of *Ppip5k2*^*+/K*^*^*, and *Ppip5k2*^*K*^*^*^*/K*^*^* mice, which coincide with the high-frequency HL observed at this age in both genotypes.

To determine whether the auditory deficit in *Ppip5k2* ^*K*^*^*^*/K*^*^* mice was secondary to hair cell loss, spiral ganglion degeneration or stria vascularis atrophy, we analyzed whole mounts preparations as well as serial sections of the cochlear tissue at P150 (Fig 6). The cytoarchitecture and morphology of the cochlea in *Ppip5k2* ^*K*^*^*^*/K*^*^* mice appear normal at P150. No obvious degeneration of inner (IHCs) and outer (OHCs) hair cells was observed in the apical and middle cochlear turn (Fig 6). However, consistent with high frequency hearing loss observed at P150, a greater degree of degeneration of OHCs in the basal coil was evident in *Ppip5k2*^*+/K*^*^* and *Ppip5k2*^*K*^*^*^*/K*^*^* mice (Fig 6). Although no difference was observed in the number of IHCs, a statistically significant (p<0.01) reduction in OHCs was observed in the basal turn (Fig 5B). No obvious indications of stria vascularis atrophy or spiral ganglion neuron degeneration were found in *Ppip5k2*^*K*^*^*^*/ K*^*^* mice at P150 (S6 Fig). Thus, the degeneration of OHCs is likely contributing to the progressive, high frequency hearing deficit in these *Ppip5k2* ^*K^/K^*^ mice. Overall, our data indicate that genetic perturbation of PP-IP turnover has consequences for cellular signaling that promotes an auditory deficit, in both humans and mice.

## Discussion

This study points to the importance of PP-IP signaling and the role of the bifunctional PP-IP kinase/phosphatase, PPIP5K2, for normal mammalian inner ear function. This conclusion is based upon a novel single variant [c.2510G>A, p. (Arg837His)] in the *PPIP5K2* gene that we associated with autosomal recessive, nonsyndromic, prelingual sensorineural deafness. Biochemical analyses revealed that, compared to WT human PPIP5K2, the PPIP5K2^R837H^ variant exhibited lower phosphatase activity and higher kinase activity, indicating it promotes increased metabolic flux from 5-IP7 to IP8 *in vivo*. The availability of a gene-trap murine model for elevated PPIP5K2 kinase activity is consistent with the association of p.(Arg837His) with an auditory deficit.

Humans who are homozygous for the c.2510G>A variant failed transient evoked otoacoustic emission tests, indicating an outer hair cell functional defect. Thus, it is significant that, in the *Ppip5k2*^*K*^*^*^*/K*^*^* mice, we found that hearing loss is associated with selective degeneration of cochlear outer hair cells. How might the latter phenomenon be promoted by elevated IP8 levels? One possibility is that hair cell loss is due to apoptosis, since previous work has shown that increased PP-IP synthesis is pro-apoptotic [33]. Another possibility emerges from recent work showing that loss of outer hair cells can result from bioenergetic imbalance [34]; the latter could also result from perturbation to PP-IP turnover and signaling [11, 12]. Deficits in vesicle trafficking and endocytosis might affect the function of the inner hair cell ribbon synapse [35, 36], and thus contribute to hearing loss observed in the *Ppip5k2*^*K*^*^* mice.

It is intriguing that the hearing deficit in the *Ppip5k2*^*K*^*^*^*/K*^*^* mice is less severe than that observed in humans homozygous for the p.(Arg837His) variant. Perhaps in mice there is greater functional redundancy of PPIP5K2 with other proteins. The particular susceptibility of humans to a single arginine-to-histidine substitution in PPIP5K2 may also reflect genetic or environmental factors. Elucidation of the causes of this dissimilarity may reveal molecular or cellular pathways for potential interventions to prevent HL in DFNB100 families. Future studies with mice in which the *Ppip5k2* gene is targeted on a genetic background resistant to age-related HL, may also help in deciphering the roles of PP-IPs in development, maintenance and function of sensory cells in the inner ear.

PPIP5Ks are pivotal enzymes for regulating PP-IP turnover. Their actions at the interface of cell signaling and bioenergetic homeostasis can impact many biological processes [11]. Yet surprisingly, as far as we are aware, our work provides the first description of any amino acid variant in either PPIP5K1 or PPIP5K2 that is both functionally-significant and associates with a human disorder. Two candidate single nucleotide polymorphisms, *rs35671301* (p.Ser419Ala) and *rs17155147* (p.Thr1267Met), in human PPIP5K2 were reported to be enriched in individuals with autism spectrum disorder [37, 38]. However, in those studies, there is no empirical or *in silico* evidence that either amino-acid replacement might alter affect protein function. Thus, PPIP5K2 is given new clinical significance by our observations.

In conclusion, the identification of the PPIP5K2 variant as a risk factor for deafness raises the possibility that there may be other variants in its coding sequence that exhibit a similar hearing deficit. Moreover, our study reinforces the concept that studies into PP-IP turnover and signaling are relevant to human health and well-being.

## Materials and methods

### Family enrollment and clinical evaluation

This study was conducted under IRB-approved protocols, by IRB committees at University of Maryland School of Medicine (UMSOM), USA (HP-00059851), National Centre of Excellence in Molecular Biology, Lahore, Pakistan (FWA00001758), and the National Institutes Health, USA (Combined Neuroscience Blue Panel IRB; OH-93-N-016), and in accord with the Declaration of Helsinki for the release of clinical information, family history, and blood draw. Written informed consent was obtained from all adult participants and parents of minor subjects. Genomic DNA was extracted from blood samples. Clinical histories were obtained for all the individuals of families PKDF041 and PKDF751. A general physical examination was performed to evaluate general health. Pure tone audiometry tests for air and bone conduction were performed at frequencies from 250 to 8,000 Hz. Tympanometry and otoacoustic emission tests were performed to assess middle ear and cochlear function. Vestibular function was evaluated by testing tandem gait ability and by using the Romberg test.

### Exome sequencing and segregation analysis

Exome sequencing on the genomic DNA of one affected individual of family PKDF751 was performed and analyzed as described previously [39].

We used the XHMM and CoNIFER methods [40, 41] to call CNV events in WES data. The segregation of the candidate variants in participating member of family PKDF751 was performed through Sanger sequencing. Primers were designed with Primer3 software, and used to amplify exons as well as flanking introns and untranslated regions (S4 Table).

### *Ppip5k2* gene-trap mouse model

All experiments were approved by the Animal Care and Use Committees at the University of Maryland, School of Medicine in accordance with the National Institutes of Health (NIH) Guide for the Care and Use of Laboratory Animals. The *B6N(Cg)-Ppip5k2*^*tm1a(EUCOMM)Wtsi/J*^ strain was generated by KOMP. In this study, we designated this strain as *Ppip5k2*^*K*^*^* due to its intact kinase domain. The *Ppip5k2*^*K*^*^* mice had a gene-trap cassette downstream of exon 13, which is expected to cause premature truncation.

### Protein expression and purification

Lipofectamine 3000 (Life Technologies) was used to transiently transfect HEK293 cells with pDest515 plasmids hosting cDNAs encoding Flag-tagged versions of either full-length PPIP5K2 (isoform 11; accession number NM_001345875), PPIP5K2 p.Arg837His (PPIP5K2^R837H^) or a truncated version of PPIP5K2 containing residues 1 to 466 (PPIP5K2^1-466^). The mutations were created using a Q5 site-directed mutagenesis kit (New England Biolabs). The primers used are as follows (mutagenic codons underlined): R837H mutation, forward: GTCTATTCTT**CAC**TATGGTGCCTTATG; reverse: AGCAAAGAATGTACATGAC. Truncation (stop codon insertion), forward: **TAG**GTCATTTTTCTGGAATAAATCG; reverse: **TTA**CTCTAATACAGTCTTAAGTTG. All mutations were confirmed by sequencing.

In some experiments, the wild type (WT) and mutant PPIP5K2 constructs were expressed in *PPIP5K*^-/-^ HEK293 cells that had been radiolabeled by growing them in media containing [^3^H]-inositol. Cellular levels of [^3^H]IP7 and [^3^H]IP8 were determined by HPLC [12].

For the purification of WT and mutant PPIP5K2 proteins, the HEK293 cells hosting the various constructs were harvested 16–20 hr after transfection and lysed in ice-cold buffer containing 10 mM HEPES, 130 mM NaCl, 1% Triton X-100, 10 mM NaF, 10 mM Na_2_HPO_4_, 10 mM Na pyrophosphate and protease inhibitor cocktail (Roche). All subsequent steps were performed on ice in an anaerobic chamber (Bactron CAT180). The FLAG-tagged PPIP5K2 proteins were immunopurified using FLAG M2 affinity gel (Sigma). Purified FLAG-PPIP5K2 proteins were analyzed by SDS-PAGE and stained with Coomassie Blue. Densitometry analysis (ImageJ) was used to calculate the amounts of PPIP5K2 loaded onto the gel, by reference to additional lanes loaded with known amounts of the human recombinant PPIP5K2 kinase domain.

### Phosphatase and kinase assays

All enzyme assays were performed using physiologically relevant substrate concentrations at 37°C; the extent of substrate conversion was up to 30%. IP8 phosphatase activities were determined using approximately 70 ng of purified, WT or mutant PPIP5K2s in 30 min incubations with100 ul of assay buffer containing 1 mM Na_2_EDTA, 50 mM KCl, 20 mM HEPES pH 7.2, 2 mM MgCl_2_, 0.5 mg/ml BSA and 1 μM [^3^H]IP8. The 5-IP7 kinase activities were determined using approximately 35 ng of these proteins in 90 min incubations with 100 ul of assay buffer containing 1 mM Na_2_EDTA, 50 mM KCl, 20 mM HEPES pH 7.2, 7 mM MgCl_2_, 5 mM ATP, 0.5 mg/ml BSA and 1 μM 5-[^3^H]IP7. All assays were then acid-quenched, neutralized, and HPLC was used to determine the degree of metabolism [12].

### Confocal imaging

Inner ear tissues were harvested from temporal bones of the WT and *Ppip5k2*^*K*^*^* mice, fixed and processed for immunostaining as previously described [42]. The following primary antibodies were used in our studies: anti-Myosin VIIa (1:200; Proteus BioSciences, Ramona, CA), anti-PPIP5K2 (1:200; Stock# ab87054, Abcam, Cambridge, MA) and anti-PPIP5K1 (1:200; Novus Biologicals, Littleton, CO). Samples were mounted using ProLong Gold antifade reagent (Life Technologies, Carlsbad, CA) and imaged using an LSM 510 DUO confocal microscope (Zeiss Microimaging Inc., Thornwood, NY) with a ×63, 1.4 N.A. oil immersion objective.

### Assessment of hearing function

Briefly, mice were anesthetized with an IP injection of ketamine/xylazine mix at 80–100 mg/kg and 10–12.5 mg/kg, body weight respectively. ABR recordings were performed using a TDT RZ6/BioSigRZ system along with RA4PA preamplifier/digitizer. The system was calibrated between 4–40KHz frequency range from 90 to 5 db using the ACO Pacific type 7016 microphone. For simultaneous recording from both ears, two open filed speakers were placed 10 cm apart from each ear. Two electrodes were placed behind each ear. The reference was placed at the base of the skull, whereas the ground was placed at the base of the tail. Mice were tested at broad band clicks and pure tone frequencies of 8, 16, 24, and 32 kHz for ABRs (n = 7 per genotype at each time point). All the stimuli were tested from 90 dB SPL to10 dB SPL in 5 dB decrements, with a total of 512 responses averaged at each level. Results are displayed as a mean and standard error of the mean (SEM). When no ABR signals were observed at 90db, for calculation purposes, mice were assigned a threshold of 95db. Statistical significance was determined using a 2-way ANOVA. A Bonferroni correction was performed to adjust for multiple statistical tests.

### RNA extraction and quantitative real-time PCR

The whole inner ear was isolated from P150 WT and *Ppip5k2*^*K*^*^*^*/K*^*^* mice using the RiboPure RNA isolation kit (Life Technologies, Grand Island, NY). cDNA was prepared using an oligo-dT primer and SMARTScribe Reverse Transcriptase enzymes (Clontech, Mountain View, CA). To determine the differential expression of *Ppip5k1*, SYBRGreen based real-time primers were designed using Integrated DNA Technologies online PrimeTime qPCR assay design tool. The real-time PCR assays were performed in triplicate using an ABI StepOnePlus Real-Time thermal cycler (ABI, Foster City, CA). CT values were normalized using *Gapdh* as an endogenous control, and fold changes of *Ppip5k1* transcripts in different tissues were calculated using 2^-ΔΔCt standard formula. An expression with a 2-fold change and with a P-value less than 0.05 based on a Student’s t-test analysis was considered significant.

### Web resources

The URLs for data presented herein are as follows:

NHLBI Exome Sequencing Project (Exome Variant Server), http://evs.gs.washington.edu/EVS/

Primer3, http://frodo.wi.mit.edu/primer3

PrimeTime qPCR assay design tool, http://www.idtdna.com/Scitools/Applications/RealTimePCR/

gEAR, http://umgear.org

SHIELD (Shared Harvard Inner-Ear Laboratory Database) https://shield.hms.harvard.edu/

## Supporting information

S1 Fig The *DFNB100* locus on chromosome 5q13.2-q23.2. Centromeric region of human chromosome 5q includes three deafness loci *DFNB100*, *DFNB49*, *USH2C*. Regions of homozygosity for each DFNB100 family are represented by vertical lines while a bar at the ends of vertical lines indicate meiotic recombinations. Note that linkage region of *DFNB100* overlaps with *DFNB49* and *USH2C* linkage intervals. Location of STR markers are based on the human Marshfield genetic map. Some candidate genes are also shown.
(TIF)

S2 Fig Anti-PPIP5K2 and anti-PPIP5K1 antibodies validations. Although we cannot rule out *in vivo* cross reactivity. However, *in vitro* both antibodies are protein-specific. (A) Immunofluorescence signal of anti-PPIP5K2 antibody coincides with the signal produced by GFP-tagged WT PPIP5K2 (top panel), and p.Arg837His variant harboring PPIP5K2 (middle panel) expressed in COS7 cells. No cross-reactivity of this antibody was detected with fluorescently tagged PPIP5K1 (bottom panel). (B) Similarly, immunofluorescence signal of anti-PPIP5K1 antibody coincides with the signal produced by GFP-tagged PPIP5K1, but not with fluorescently tagged PPIP5K2 expressed in COS7 cells. (C) The anti-PPIP5K2 antibody was further tested by western blot analysis in HEK293 cells lysate transfected with EGFP vector, GFP-tagged WT and mutant PPIP5K2 expression constructs. The anti-PPIP5K2 antibody detected the specific bands of sizes corresponding to full-length PPIP5K2 ~140 kDa. Anti-GFP antibodies were used as loading control. (D) Specificity of the anti-PPIP5K2 antibodies were further validated by western blot on whole protein lysates from heart, kidney and liver tissues from *Ppip5k2*^*+/K*^*^* and *Ppip5k2*^*K*^*^*^*/K*^*^* mice. Protein products of expected size were observed in the heart and liver samples from *Ppip5k2*^*+/K*^*^* mice, which were absent in lysates from the *Ppip5k2*^*K*^*^*^*/K*^*^* mice tissues.
(TIF)

S3 Fig Murine PPIP5K1 and PPIP5K2 have similar expression profiles. (A) The expression of *Ppip5k1* is detectable in all the organs tested by real-time qPCR. Expression was normalized against the house-keeping gene *Gapdh* (ΔCT) and shown relative to expression in liver (ΔΔCT). (B) Cross-section through one of the coils of inner ear showing diffuse cytoplasmic immunolabeling of PPIP5K1 throughout the cochlear duct, including the organ of Corti (C), spiral ganglion neurons (D), and stria vascularis (E). PPIP5K1 expression pattern in the cochlear tissue is very similar, but is relatively weaker than PPIP5K2. Scale bars: 100μm (panel B), and 10μm (panels C-E). (F) In *Ppip5k2*^*K*^*^*^*/K*^*^* mice, *Ppip5k1* expression is upregulated, with more than two-fold increase in the inner ear, when compared with expression for same tissue type from WT mice. For each tissue sample *Ppip5k1* expression was normalized to *Gapdh* and shown as fold change relative to WT expression.
(TIF)

S4 Fig SDS-PAGE gel showing the purified recombinant PPIP5K2^WT^, PPIP5K2^R837H^ and PPIP5K2^1-466^ proteins used for functional studies. Arrows indicate the position of the various PPIP5Ks and also standards used for quantifying the proteins.
(TIF)

S5 Fig Representative ABR wave forms from control and mutant mice at various developmental stages. (TIF)

S6 Fig No obvious degeneration of spiral ganglion neurons or stria vascularis was observed in *Ppip5k2*^*+/K*^*^* and *Ppip5k2*^*K*^*^*^*/K*^*^* mice at P150. (A) Cochlear cross-section of *Ppip5k2*^*+/+*^, *Ppip5k2*^*+/K*^*^*, and *Ppip5k2*^*K*^*^*^*/K*^*^* mice exhibit no gross difference between genotypes. (B) Histological analysis of the apical (left panel), middle (middle panel) and basal turns (right panel) of the cochlea from P150 mice show no apparent spiral ganglion neurons or stria vascularis degeneration in all three cochlear turns. Scale bar: 200μm.
(TIF)

S1 Table Whole exome sequencing filtration scheme. (DOCX)

S2 Table Bioinformatics evaluation of deafness-associated variants found in *PPIP5K2*. (DOCX)

S3 Table SNP genotypes in 1,885,410 bp flanking *PPIP5K2* variant. (DOCX)

S4 Table Primer sequences used to amplify and sequence human *PPIP5K2* coding exons and splice junctions. (DOCX)
