# Supplementary figures and images for: Mutations in Diphosphoinositol-Pentakisphosphate Kinase PPIP5K2 are associated with hearing loss in human and mouse
Source: PLoS Genet. 2018 Mar 28;14(3):e1007297. doi: 10.1371/journal.pgen.1007297 (PMC5891075; doi:10.1371/journal.pgen.1007297)

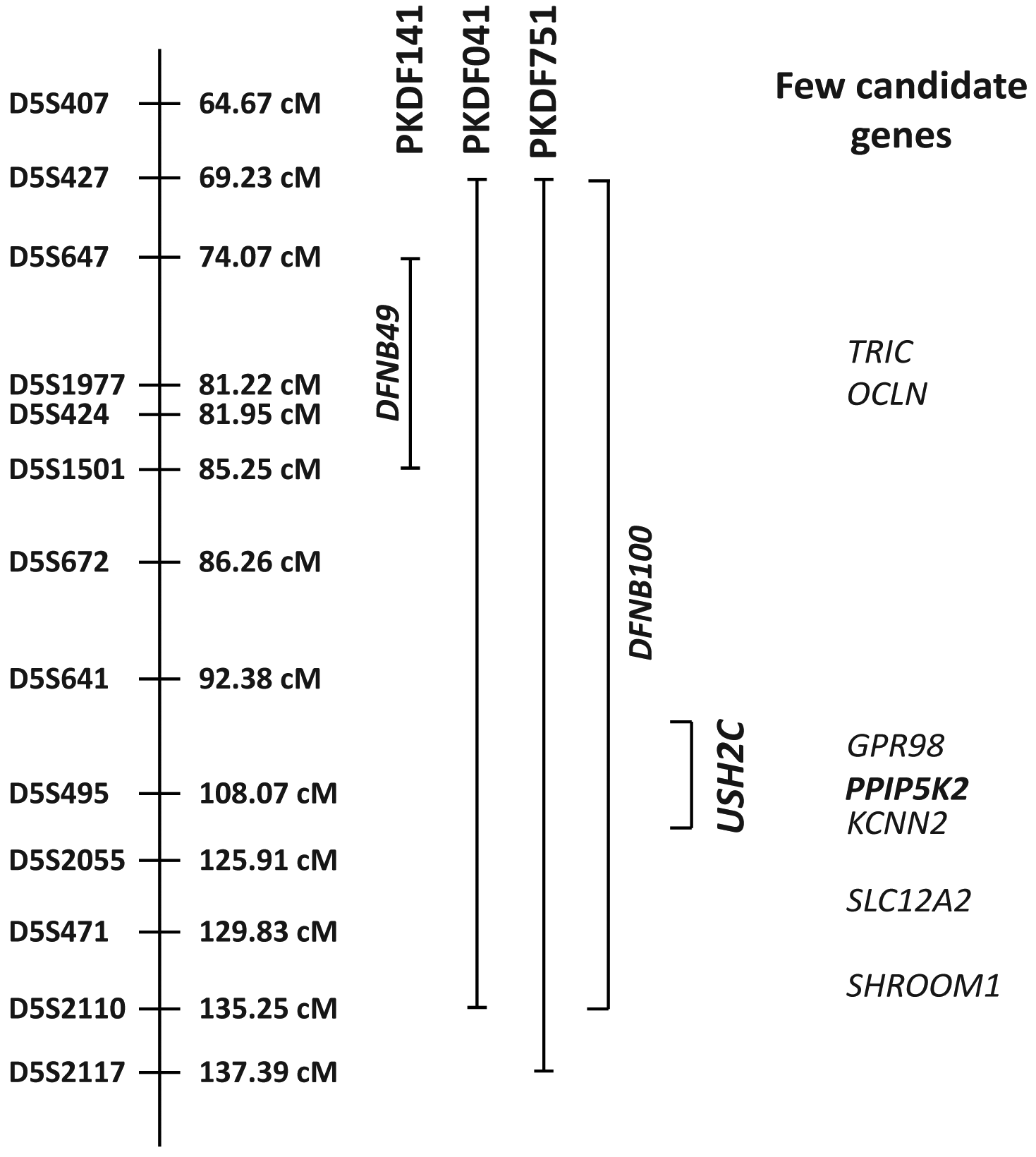

Supplement: S1 Fig — Centromeric region of human chromosome 5q includes three deafness loci DFNB100, DFNB49, USH2C. Regions of homozygosity for each DFNB100 family are represented by vertical lines while a bar at the ends of vertical lines indicate meiotic recombinations. Note that linkage region of DFNB100 overlaps with DFNB49 and USH2C linkage intervals. Location of STR markers are based on the human Marshfield genetic map. Some candidate genes are also shown. (TIF) [file pgen.1007297.s001.tif]

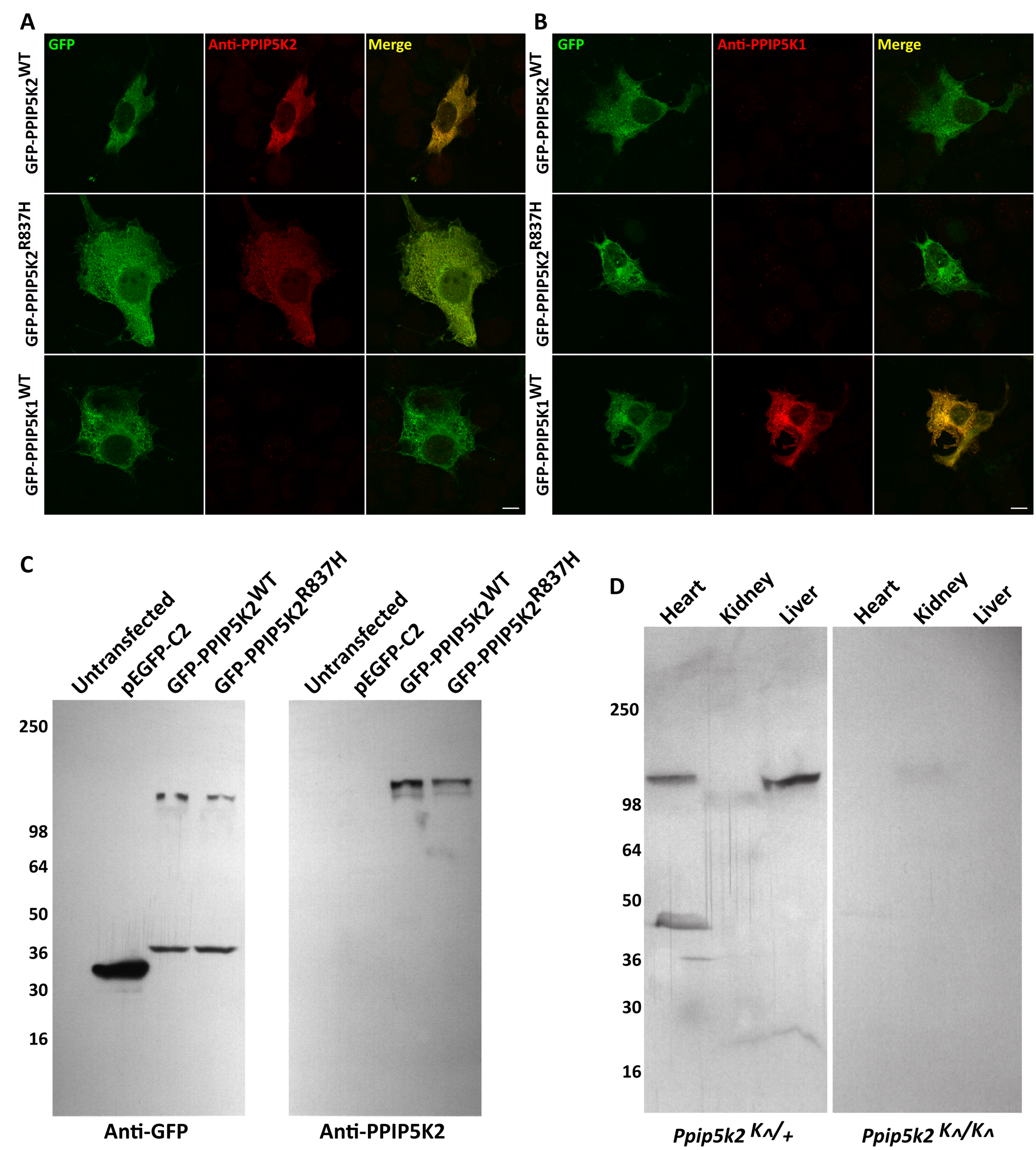

Supplement: S2 Fig — Although we cannot rule out in vivo cross reactivity. However, in vitro both antibodies are protein-specific. (A) Immunofluorescence signal of anti-PPIP5K2 antibody coincides with the signal produced by GFP-tagged WT PPIP5K2 (top panel), and p.Arg837His variant harboring PPIP5K2 (middle panel) expressed in COS7 cells. No cross-reactivity of this antibody was detected with fluorescently tagged PPIP5K1 (bottom panel). (B) Similarly, immunofluorescence signal of anti-PPIP5K1 antibody coincides with the signal produced by GFP-tagged PPIP5K1, but not with fluorescently tagged PPIP5K2 expressed in COS7 cells. (C) The anti-PPIP5K2 antibody was further tested by western blot analysis in HEK293 cells lysate transfected with EGFP vector, GFP-tagged WT and mutant PPIP5K2 expression constructs. The anti-PPIP5K2 antibody detected the specific bands of sizes corresponding to full-length PPIP5K2 ~140 kDa. Anti-GFP antibodies were used as loading control. (D) Specificity of the anti-PPIP5K2 antibodies were further validated by western blot on whole protein lysates from heart, kidney and liver tissues from Ppip5k2+/K^ and Ppip5k2K^/K^ mice. Protein products of expected size were observed in the heart and liver samples from Ppip5k2+/K^ mice, which were absent in lysates from the Ppip5k2K^/K^ mice tissues. (TIF) [file pgen.1007297.s002.tif]

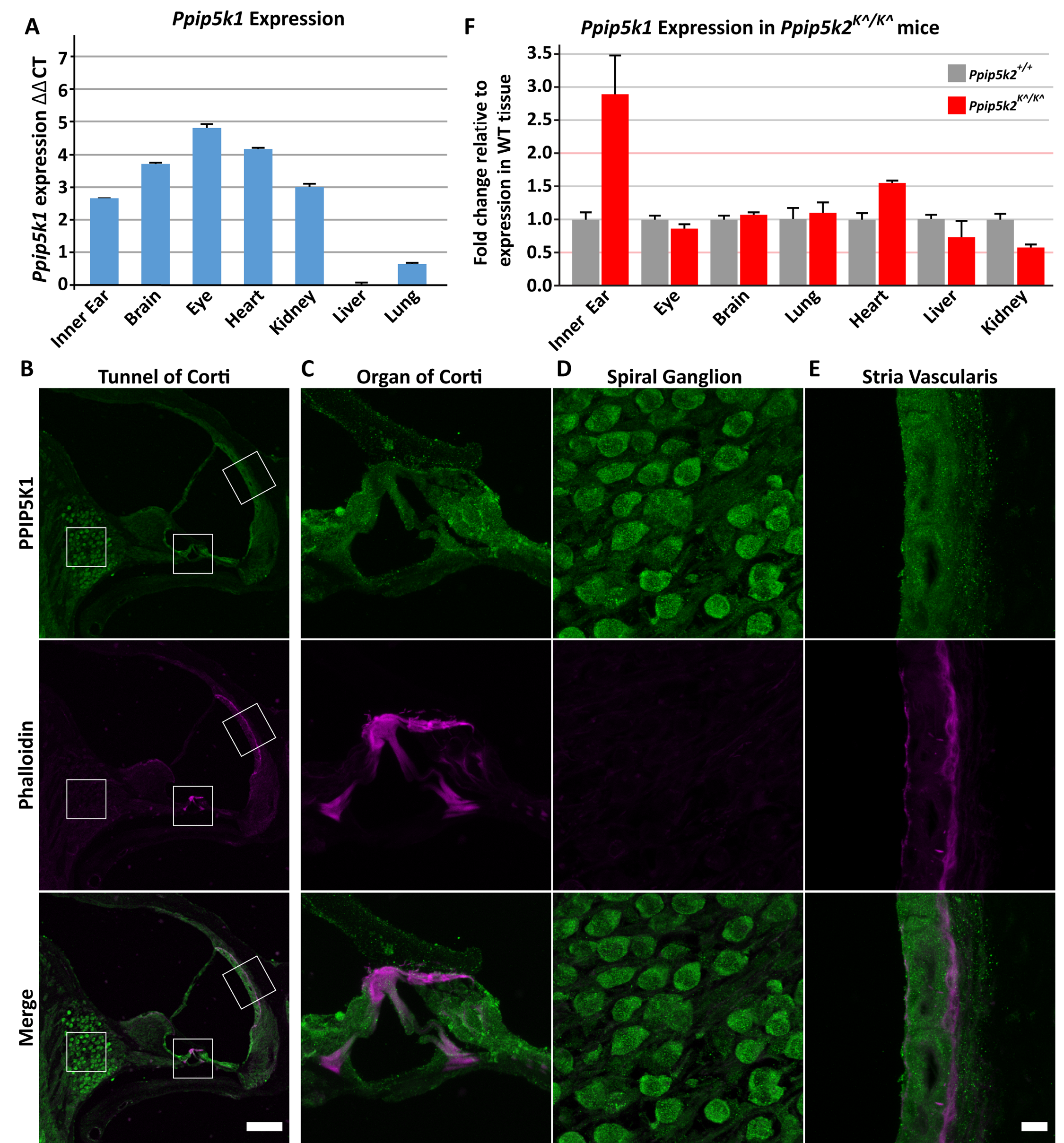

Supplement: S3 Fig — (A) The expression of Ppip5k1 is detectable in all the organs tested by real-time qPCR. Expression was normalized against the house-keeping gene Gapdh (ΔCT) and shown relative to expression in liver (ΔΔCT). (B) Cross-section through one of the coils of inner ear showing diffuse cytoplasmic immunolabeling of PPIP5K1 throughout the cochlear duct, including the organ of Corti (C), spiral ganglion neurons (D), and stria vascularis (E). PPIP5K1 expression pattern in the cochlear tissue is very similar, but is relatively weaker than PPIP5K2. Scale bars: 100μm (panel B), and 10μm (panels C-E). (F) In Ppip5k2K^/K^ mice, Ppip5k1 expression is upregulated, with more than two-fold increase in the inner ear, when compared with expression for same tissue type from WT mice. For each tissue sample Ppip5k1 expression was normalized to Gapdh and shown as fold change relative to WT expression. (TIF) [file pgen.1007297.s003.tif]

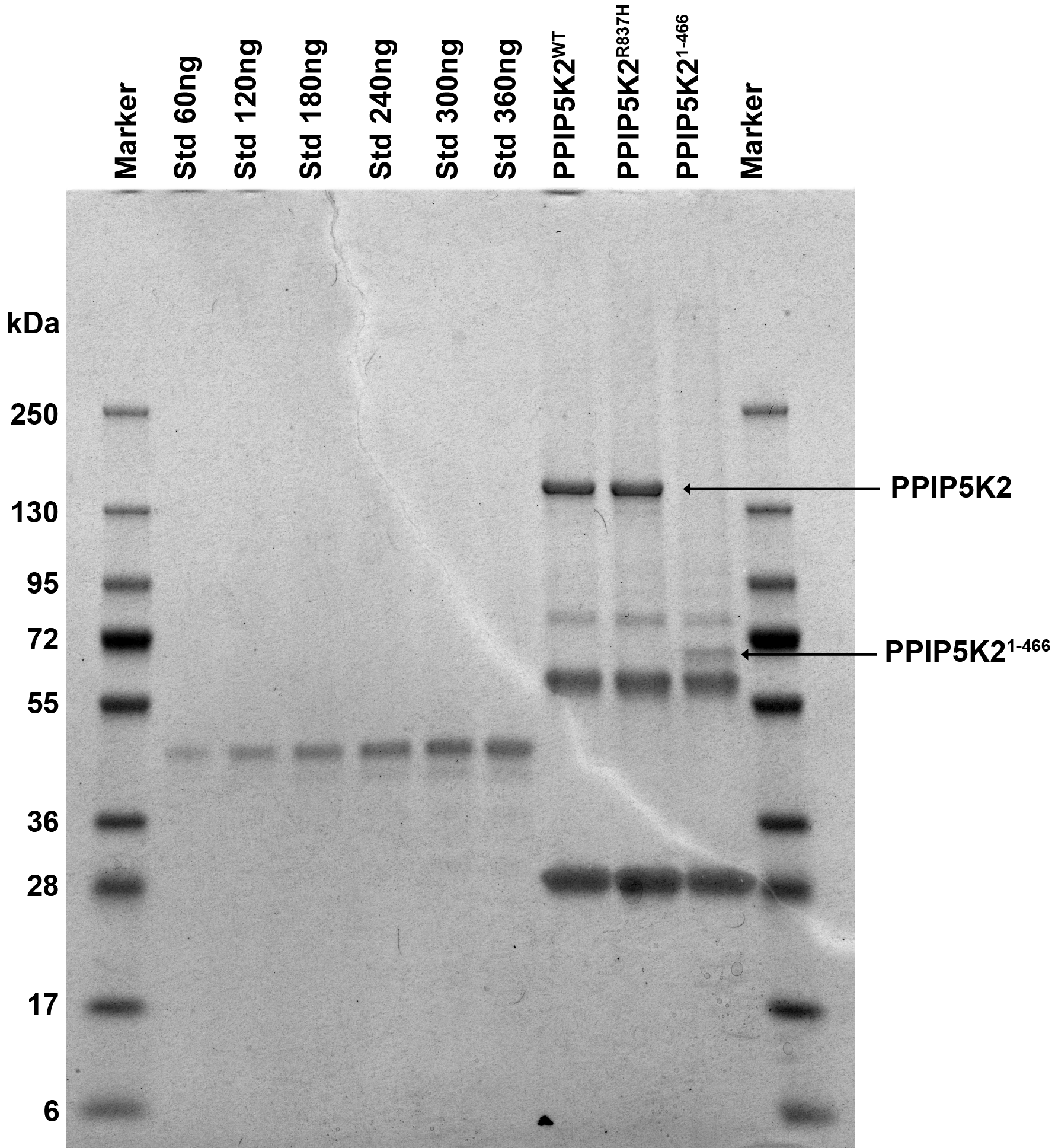

Supplement: S4 Fig — Arrows indicate the position of the various PPIP5Ks and also standards used for quantifying the proteins. (TIF) [file pgen.1007297.s004.tif]

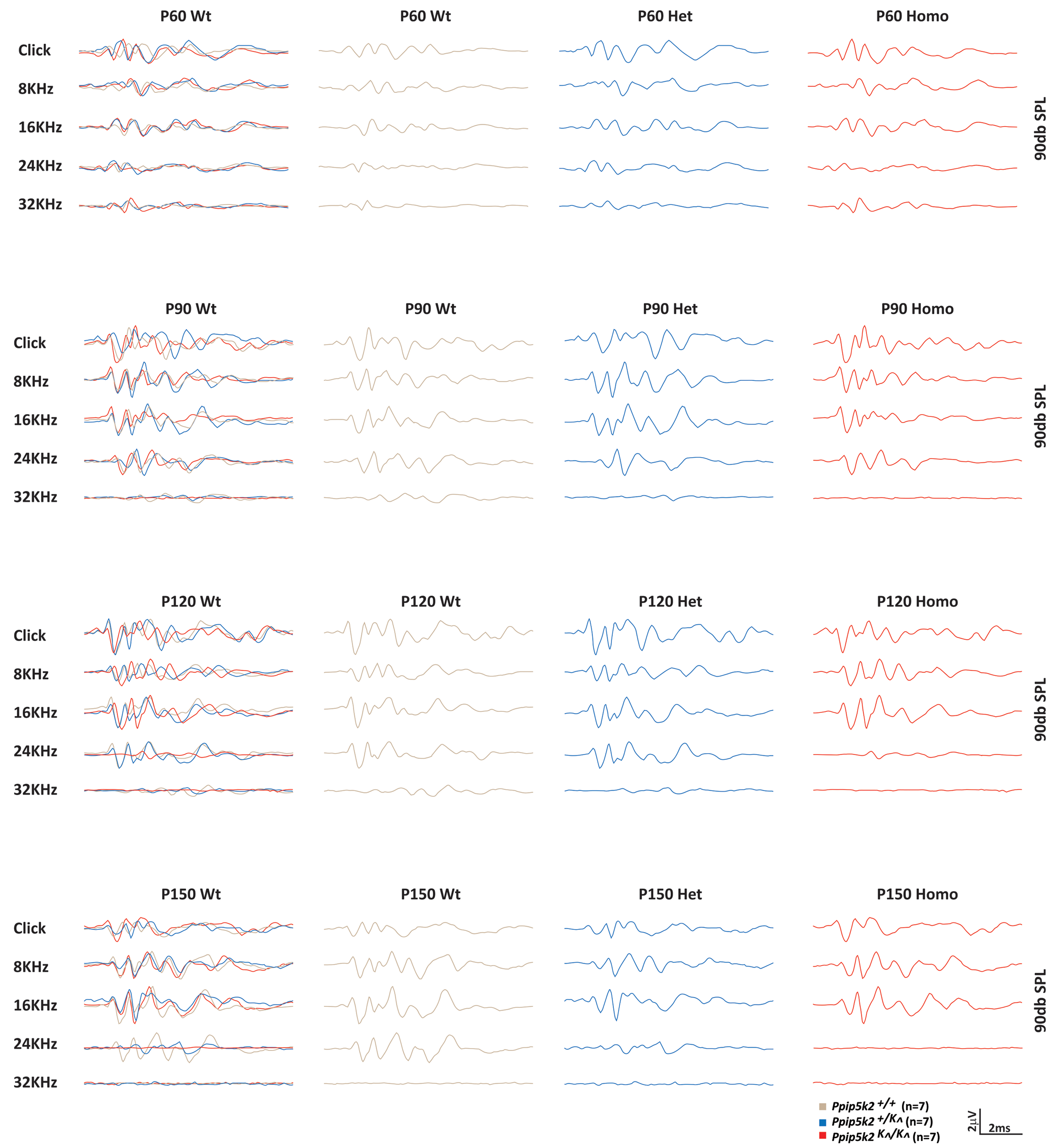

Supplement: S5 Fig — (TIF) [file pgen.1007297.s005.tif]

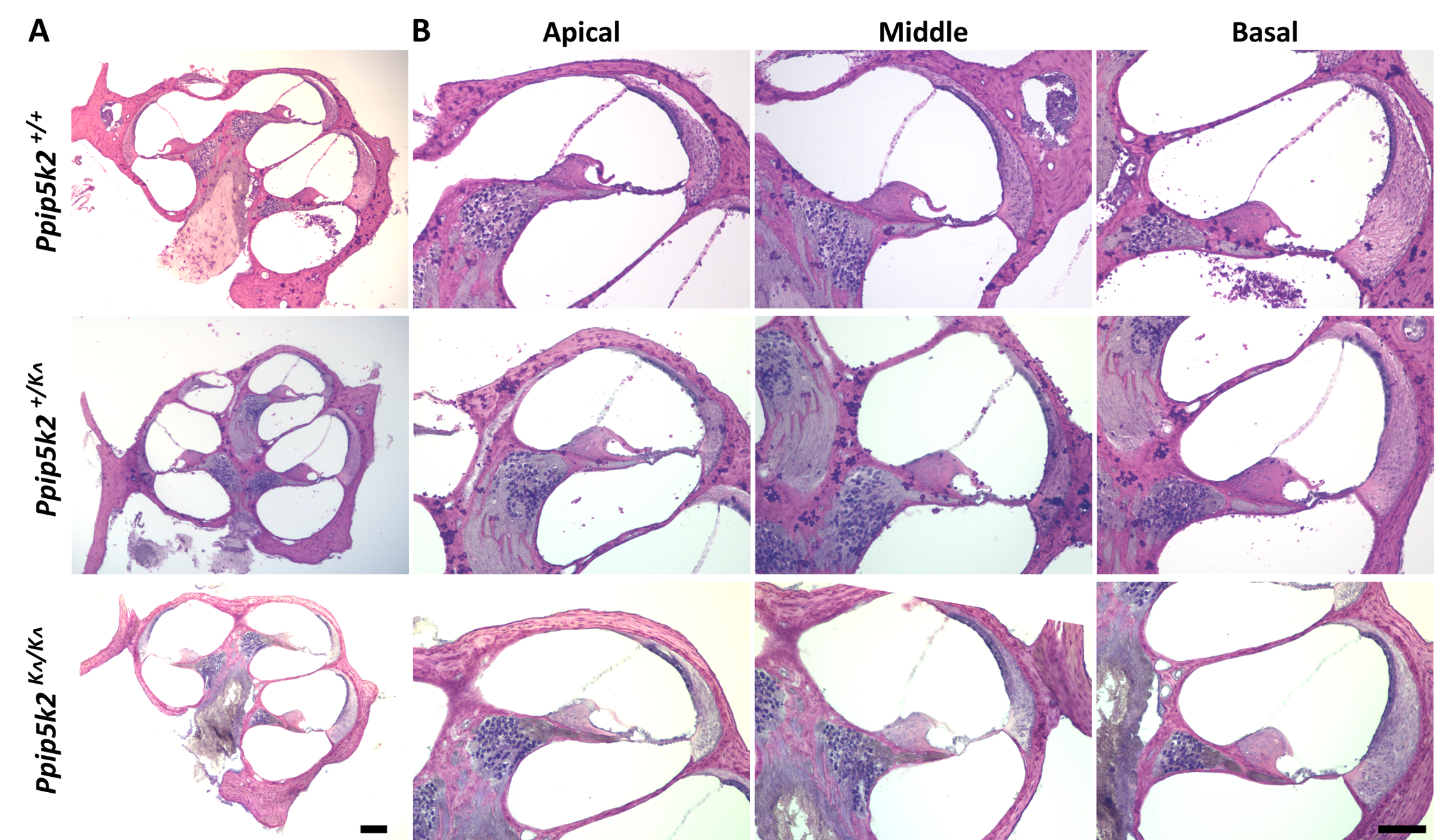

Supplement: S6 Fig — (A) Cochlear cross-section of Ppip5k2+/+, Ppip5k2+/K^, and Ppip5k2K^/K^ mice exhibit no gross difference between genotypes. (B) Histological analysis of the apical (left panel), middle (middle panel) and basal turns (right panel) of the cochlea from P150 mice show no apparent spiral ganglion neurons or stria vascularis degeneration in all three cochlear turns. Scale bar: 200μm. (TIF) [file pgen.1007297.s006.tif]
