# Supplementary material for: Mutations in Diphosphoinositol-Pentakisphosphate Kinase PPIP5K2 are associated with hearing loss in human and mouse
Source: PLoS Genet. 2018 Mar 28;14(3):e1007297. doi: 10.1371/journal.pgen.1007297 (PMC5891075; doi:10.1371/journal.pgen.1007297)
Supplement: S1 Table — (DOCX) [file pgen.1007297.s007.docx]

**Table S1. Whole exome sequencing filtration scheme**

| Filters | Affected Exome Variants |
| --- | --- |
| Total changes* | 116,944 |
| Non-Syn/SS/ins/del^#^ | 8,575 |
| Not present in control | 1,716 |
| Homozygous/compound heterozygous changes | 678 |
| Changes with > 1% allele frequency | 22 |
| Changes <0.001% allele frequency in 1000 genome/NHLBI exome/ExAC databases | 6 |
| Genes with predicted pathogenic changes^†^ | 3 |
| Potential pathogenic changes in known deafness genes | 0 |
| Changes present in all the affected individuals^‡^ | 1 |

***** Changes shown with minimum 10X coverage

**^#^** Non-Syn: non-synonymous; SS: splice site; ins: insertion; del: deletion

**^†^** Predicted pathogenic with at least two programs

**^‡^** Segregation analysis among the affected individuals through Sanger sequencing
