# Supplementary material for: Mutations in Diphosphoinositol-Pentakisphosphate Kinase PPIP5K2 are associated with hearing loss in human and mouse
Source: PLoS Genet. 2018 Mar 28;14(3):e1007297. doi: 10.1371/journal.pgen.1007297 (PMC5891075; doi:10.1371/journal.pgen.1007297)
Supplement: S2 Table — (DOCX) [file pgen.1007297.s008.docx]

**Table S2. Bioinformatics evaluation of variants found in *PPIP5K2***

| **Bioinformatics Tools** | ***PPIP5K2*** |
| --- | --- |
| hg38 coordinates | Chr5:103173953 |
| Nucleotide accession number | NM_001276277 |
| Nucleotide mutation | c.2510G>A |
| Protein accession number | NP_001263206 |
| Amino acid substitution | p.Arg837His |
| dbSNP 147 | rs548137246 |
| ExAC Allele frequency | 0.0001406 |
| PhyloP ^A^ | 7.923 |
| GERP ^B^ | 5.19 |
| PolyPhen-2 | Probably Damaging (0.976) |
| Provean | Deleterious (-4.64) |
| SIFT | Damaging (0.001) |
| MutationTaster | Disease-causing |
| Mutation Assessor | Medium |
| SNPs3D | **-1.31 (Pathogenic)** |
| LRT | **Deleterious** |
| mCSM ^C^ | -1.536 Kcal/mol (Destabilizing) |
| DUET ^C^ | -1.403 Kcal/mol (Destabilizing) |

^A^ PhyloP scores indicate nucleotide conservation under a null hypothesis of neutral evolution, where conserved sites are assigned positive scores.

^B^ GERP provides position-specific estimates of evolutionary constraint. Positive scores are indicative of fewer substitutions than the average neutral site.

^C^ Predicts the effect of a single mutation on protein structure stability (∆∆G). A negative value corresponds to a mutation predicted as destabilizing.
