# Supplementary material for: Mutations in Diphosphoinositol-Pentakisphosphate Kinase PPIP5K2 are associated with hearing loss in human and mouse
Source: PLoS Genet. 2018 Mar 28;14(3):e1007297. doi: 10.1371/journal.pgen.1007297 (PMC5891075; doi:10.1371/journal.pgen.1007297)
Supplement: S3 Table — (DOCX) [file pgen.1007297.s009.docx]

**Table S3.** **SNP genotypes in 1,885,410 bp flanking *PPIP5K2* variant**

| **dbSNP build 147** | **Chromosome:Position** | **~Mb^a^** | **PKBF041** | **PKDF751** |
| --- | --- | --- | --- | --- |
| ***rs1030234*** | chr5:102119807 | 102.12 | C C | C C |
| ***rs4703215*** | chr5:102218290 | 102.22 | T T | T T |
| ***rs61566297*** | chr5:102322707 | 102.32 | G G | T T |
| ***rs1542296*** | chr5:102419726 | 102.42 | T T | T T |
| ***rs6871899*** | chr5:102521240 | 102.52 | G G | G G |
| ***rs3114600*** | chr5:102619495 | 102.62 | C C | C C |
| ***rs258256*** | chr5:102726539 | 102.73 | A A | A A |
| ***rs467897*** | chr5:102819906 | 102.82 | A A | A A |
| ***rs55641526*** | chr5:102915301 | 102.92 | G G | G G |
| ***rs17154953*** | chr5:103019557 | 103.02 | A A | A A |
| ***rs548137246***  **(*PPIP5K2 c.2510G>A*)** | chr5:103173953 | 103.17 | **A A** | **A A** |
| ***rs183707*** | chr5:103305081 | 103.31 | G G | G G |
| ***rs75237378*** | chr5:103403441 | 103.40 | A A | A A |
| ***rs291870*** | chr5:103505987 | 103.51 | T T | T T |
| ***rs11242498*** | chr5:103605044 | 103.61 | C C | C C |
| ***rs17393226*** | chr5:103703907 | 103.70 | G G | G G |
| ***rs11955253*** | chr5:103804703 | 103.80 | T T | C C |
| ***rs40054*** | chr5:103902044 | 103.90 | A A | A A |
| ***rs6893189*** | chr5:104005217 | 104.01 | G G | G G |

**^a^** Nucleotide location in megabases according to human reference sequence GRCh38/hg38.
