# Supplementary material for: Mutations in Diphosphoinositol-Pentakisphosphate Kinase PPIP5K2 are associated with hearing loss in human and mouse
Source: PLoS Genet. 2018 Mar 28;14(3):e1007297. doi: 10.1371/journal.pgen.1007297 (PMC5891075; doi:10.1371/journal.pgen.1007297)
Supplement: S4 Table — (DOCX) [file pgen.1007297.s010.docx]

**Table S4: Primer sequences used to amplify and sequence human *PPIP5K2* coding exons and splice junctions**

| **Exon** | **Forward Primer** | **Reverse Primer** | **Product (bp)^a^** |
| --- | --- | --- | --- |
| 1 | CGGTCAGAAAGGTTAGGACTGA | AGCACAGGAGACAAAGCAGAAG | 399bp |
| 2 | TGGGCTGATTTTACAAGTGTCAT | TCAACAACATCAGTCATGAAACAA | 626bp |
| 3 | TTTGGAAAACAAATGAAGATAGCTG | CCTGTCCATGAAAGTGAGTTAATGT | 395bp |
| 4 | TTTATGGGAGGTGGCATCAAT | TACGTGGGGAACTTGTGACTTT | 398bp |
| 5 | TTGAATATTCGAAGTGACATTTGTTT | TGTCAAAAGTAGGCCAAAGAAGA | 481bp |
| 6 | CATACGTATCATAAATGTTGGCACA | GCCATGCTTTTCTAACAAAGTATATCA | 499bp |
| 7 | TTGAGTTACATGCAGCAACCTG | CCCTAATCAAAAAGAAAGAGATCCA | 496bp |
| 8 | TGTGTCTACTAGCCTGGATTCTGA | CGGTTTACTTCTCCAATTATAAGCAC | 389bp |
| 9 | TGTAGCCCCTTATGCGTTATTG | GAATGCTTAAAAACCCTGATCATTT | 476bp |
| 10 | AGAATGGATTCTGTGGGTTATCTTT | AAGCTGAATCATATGAAATCATACACA | 367bp |
| 11 | CATGGGAAAGATCTTGAAGTGG | TTTGTACCCACCCACTGGTTTA | 362bp |
| 12_13 | CAATCAAAGTTGAGCAGCAACA | CACATTAGATTTTCCTTCATGTCTCA | 591bp |
| 14 | CGAAGAATTAAACAGGCGGTTA | CACCATGCCATTCCCTAAACTA | 296bp |
| 15_16 | TTCACTGCAGAAGAGAGCACAG | CCCAGCCAAAAATGTTTTAAGATAC | 555bp |
| 17 | CAGTGTCAGACCCTTGTCTCAA | ACAGAAGCCAAAGTTGTTCCAA | 581bp |
| 18 | GGGTCAACTGTATTTTGTGGTCA | GCTCTCAAGAGAGGCTTGATTTC | 392bp |
| 19 | GGTTGCTAGTAGCGTATGCCTAAA | TTCCAAGGACATGAAAACCAAC | 474bp |
| 20 | ACTCGGAGTTATCCAACGTTCA | TGCCAATGACACACTTCTGACT | 367bp |
| 21 | GGTTGGAAGAATAATAGATTTTTGTGG | TGCCACCAGTCTATAAAATCACCT | 498bp |
| 22 | CAAAGGAAATTTATCTGTGATGTACG | TTTCCCACCATCATATAGTTCATGT | 342bp |
| 23_24 | GGATTAAACAAGCTACCATAGTGACAA | TTTAAACCATTTTCCTGCTTAGAAGTT | 531bp |
| 25 | TGCCTTTTCCCTCATAGTACACA | CCAAGCTCCACAATTAAAGCTG | 451bp |
| 26 | TTCTGATCATTTGGCTCTGTTG | CTTTCCCTTGAATATTGGCAGA | 448bp |
| 27 | ACTCCCTTCAAAAGGGGATCTT | AGTGGTTATCATTGCAGGCATC | 478bp |
| 28 | GCACTTCCAAAGGTTGCCTTAT | GGCAAAAGATGACAGGATTTCA | 360bp |
| 29 | GACACTTTTCCTCTGTCATCAAGC | TTTGGGTGAATAACAAGTTGGAA | 400bp |
| 30 | TGAATTGTCATTTGACTCTTCCTTT | CCTGCTAGATTTTATAACAGGCAAA | 399bp |
| 31 | TGCATGTGATAGACTGAAAAGACC | GTCCCTTTACACTCCCCTTTGT | 396bp |
| 32 | TGCTGAATTTACCTTGACCATTTT | TACGCTCTAGGGAAGTGCAAAG | 380bp |
| 33 | CAAGAATTGTTTTGTCAATCAGCA | GCTGCTCACAGAATGCTAACAA | 399bp |

^a^All PCR products were amplified with 1.5 mM MgCl_2_ and 58^o^C annealing temperature.
